# Imaging and phase-locking of non-linear spin waves

**DOI:** 10.1038/s41467-022-32224-0

**Published:** 2022-08-23

**Authors:** Rouven Dreyer, Alexander F. Schäffer, Hans G. Bauer, Niklas Liebing, Jamal Berakdar, Georg Woltersdorf

**Affiliations:** 1 Institute of Physics, Martin Luther University Halle-Wittenberg, Von-Danckelmann-Platz 3, 06120 Halle, Germany; 2 Jahnstrasse 23, 96050 Bamberg, Germany; 3 Max Planck Institute of Microstructure Physics, Weinberg 2, 06120 Halle, Germany

**Keywords:** Magnetic properties and materials, Spintronics

## Abstract

Non-linear processes are a key feature in the emerging field of spin-wave based information processing and allow to convert uniform spin-wave excitations into propagating modes at different frequencies. Recently, the existence of non-linear magnons at half-integer multiples of the driving frequency has been predicted for Ni_80_Fe_20_ at low bias fields. However, it is an open question under which conditions such non-linear spin waves emerge coherently and how they may be used in device structures. Usually non-linear processes are explored in the small modulation regime and result in the well known three and four magnon scattering processes. Here we demonstrate and image a class of spin waves oscillating at half-integer harmonics that have only recently been proposed for the strong modulation regime. The direct imaging of these parametrically generated magnons in Ni_80_Fe_20_ elements allows to visualize their wave vectors. In addition, we demonstrate the presence of two degenerate phase states that may be selected by external phase-locking. These results open new possibilities for applications such as spin-wave sources, amplifiers and phase-encoded information processing with magnons.

## Abstract (web summary)

Many proposed spin-wave based devices make use of non-linear behaviour of spin-waves. Here, Dreyer et al show the emergence of non-linear spin waves oscillating at half-integer harmonics in the strong modulation regime. By applying super-Nyquist sampling Kerr microscopy they directly image these non-linear spin waves and demonstrate their phase-locking to an external frequency source.

## Introduction

In contrast to other common types of waves (e.g., electromagnetic or sound) the interaction of spin waves in magnetic materials is intrinsically non-linear due to dipolar coupling effects. Non-linear processes even dominate the response of magnetic materials for large excitation amplitudes^1,2^ and can be utilized to generate and amplify coherent collective excitations of the spin system—known as magnons. These non-linear processes are usually described as magnon-magnon scattering processes^3^ and act as additional decay channels for the homogeneous mode^4^.

Three and four-magnon scattering processes are ubiquitous effects due to the strong non-linearity of the equation of motion. For example, in three-magnon scattering^5,6^ typically the excitation of a uniform magnon leads to scattering into a magnon pair at half of the driving frequency with opposing wave vectors^7–9^. In addition, four magnon scattering processes^4,10–12^ may be used for harmonic generation^13–16^ or the amplification and stabilization of propagating spin waves^17^. Besides frequency conversion effects, non-linear magnon-scattering processes are potentially also suitable for short wavelength spin-wave generation. Such sources are required for highly-integrated magnonic devices^18–20^. For spin-wave based information processing well controlled propagation, as well as phase-locking, are required. Recently, non-reciprocal propagation and frequency selection of spin waves was demonstrated in magnetic hybrid structures ^21,22^. Phase-locking of multiple spin-wave sources has been achieved via propagating spin waves using spin-Hall^23,24^ or spin–torque nano-oscillators^25–28^. More generally, phase-locking in spin systems can be achieved by a variety of coupling mechanisms such as cavity photons^29^, acoustic phonons^30^, exchange spin waves^22^, or currents in a superconductor^31^. In addition, non-linear phenomena were discussed as control mechanism for coherent information processing^32^ in the emerging field of cavity magnonics^33–35^.

The theoretical description of these non-linear spin-wave (NLSW) phenomena, also known as spin-wave instabilities^36^, was established by Suhl^2,37^ and L’vov^3^. Recently, Bauer et al.^38^ presented a model for the description of NLSWs, considering the inherent frequency modulation of the excited magnons at low magnetic bias fields due to the anisotropic spin-wave dispersion. Here, the limit of large magnetization modulation can be treated and gives rise to a novel class of non-linear excitations. Specifically, strong amplitude-phase-oscillations result in parametrically generated spin waves oscillating at odd half-integer multiples of the driving frequency. Such excitations are not described by the conventional non-linear spin-wave theory. In soft ferromagnets, such as Ni_80_Fe_20_, these NLSWs are predicted to dominate at low bias fields with threshold driving fields below the ones of conventional non-linear processes^38,39^. So far, no direct evidence for the existence of such spin waves has been provided.

In this work, we demonstrate the direct phase-resolved imaging of this novel class of parametric spin-wave excitations in magnetic microstructures by using super-Nyquist sampling magneto-optical Kerr microscopy. In doing so, we identify the predicted large wave vector non-linear magnons oscillating at half-integer harmonics as the dominant excitation. Our experimental results are in agreement with theoretical predictions from our analytical model as well as with micromagnetic simulations. Moreover, by exploiting the phase sensitivity of our technique we reveal different regimes of phase stability of the parametrically generated spin-wave excitations which we link to two distinct phase states. Finally, we demonstrate that phase-locking of the parametric spin waves can be achieved revealing the potential of this phenomenon for phase-encoded information processing in magnon-based devices, such as spin-wave emitters.

## Results

### Non-linear spin-wave excitations

**Fig. 1:**
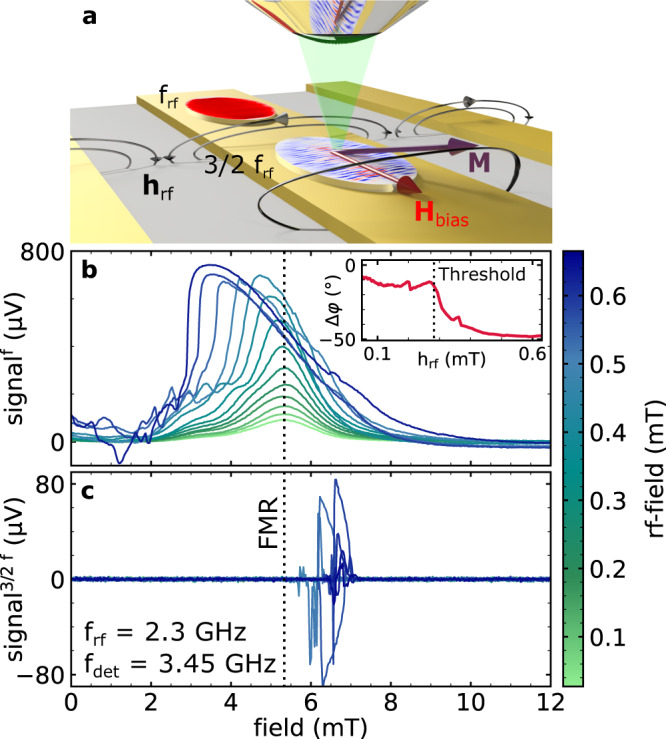
Experimental geometry and fieldswept SNS-MOKE measurements at f_rf_ and 3/2 f_rf_. In **a**, the experimental geometry is depicted. In **b** and **c**, field swept measurements with an excitation at 2.3 GHz were performed as a function of the rf-field amplitude indicated by the color code. In **b**, the imaginary part of the dynamic susceptibility detected at f_rf_ shows a typical Lorentzian line shape for low power levels with the resonance field indicated by the black dotted line. The phase shift as a function of rf-field amplitude is shown in the inset of **b**. In **c**, the NLSW response at 3/2 f_rf_ is presented.

In the experiments, we utilize super-Nyquist sampling Kerr microscopy (SNS-MOKE)^40^ to investigate coherent non-linear spin-wave excitations in 20 nm thick Ni_80_Fe_20_ elements placed on top of a coplanar wave guide (CPW), as depicted in Fig. 1a. At low bias fields, the rf-magnetic field generated by the CPW causes magnetization precession with large ellipticity. For low rf-driving field amplitudes the imaginary part of the dynamic susceptibility results in a Lorentzian resonance line shape as a function of the external bias field, as demonstrated in Fig. 1b for the case of a 30 μm × 15 μm elliptical Ni_80_Fe_20_ element. As the excitation amplitude is increased, the in-plane excursion angle of the magnetization becomes very large (of the order of 20°)^41^. The elliptical precession trajectory caused by the in-plane shape anisotropy results in an inherent frequency modulation due to the anisotropic nature of the spin-wave dispersion. A good example where these conditions are satisfied is a 20 nm thick Ni_80_Fe_20_ layer, as predicted in ref. 38. For large driving amplitudes the phase of the response changes as soon as a threshold rf-field of ≈0.30 mT is exceeded (inset in Fig. 1b). At the same time the resonance condition shifts towards lower magnetic bias fields^41^.

**Fig. 2:**
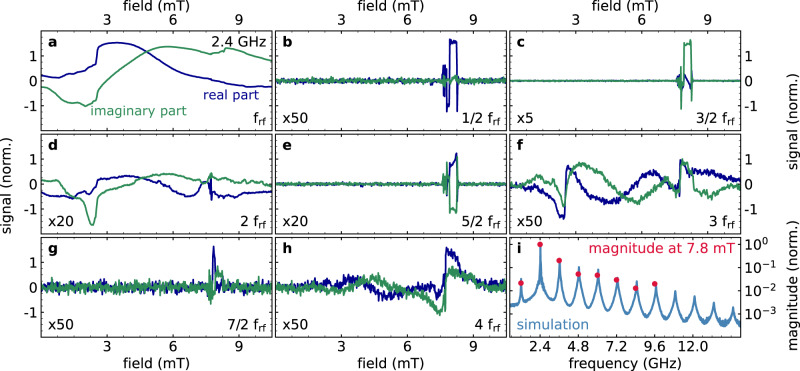
Magneto-optical sampling of non-linear spin waves at integer and half-integer harmonics. The 30 μm × 15 μm elliptical element is excited with a large rf-amplitude at an rf-frequency of 2.4 GHz and the non-linear magnetization dynamics are analyzed at different harmonics of the driving frequency as seen in **b**–**g**. The real and imaginary part of the uniform mode at f_rf_ normalized to its magnitude is presented in **a** while integer harmonics at 2 f_rf_, 3 f_rf_, and 4 f_rf_ are shown (with corresponding scaling factors) in **d**, **f**, and **h**, respectively. For large driving amplitudes coherent half-integer multiples at 1/2 f_rf_, 3/2 f_rf_, 5/2 f_rf_, and 7/2 f_rf_ become visible in a narrow field range around 7.8 mT as presented in **b**, **c**, **e**, and **g**, respectively. **i** Depicts the experimentally obtained magnitude analyzed at a fixed field value of 7.8 mT for all shown harmonics (red) while the solid line corresponds to the frequency spectrum obtained from corresponding micromagnetic simulations (blue).

In order to capture the physics of non-linear spin waves excited at low bias fields and large driving amplitudes, it is necessary to treat the magnetization dynamics explicitly within the limit of large modulation. For this, the Landau–Lifshitz–Gilbert equation can be considered in k-space. Using algebraic transformations, it assumes the form of well-known Mathieu or Hill equations. The advantage of this procedure is that the critical spin-wave modes oscillating at half-integer multiples of the pumping frequency can be quickly and reliably identified numerically^38^. The obtained solutions for the critical spin waves are characterized by strong amplitude-phase-oscillations. These amplitude-phase-oscillations are connected with frequency components at several odd half-integer multiples of the driving frequency. Our aim is now to directly image these spin waves and to resolve their frequency components. For this, a phase-stable non-linear response is required. In comparison to more common magneto-optical imaging approaches which are typically sensitive to the magnitude of the magnetic excitation, the SNS-MOKE technique provides a unique combination of phase-resolved lock-in detection while offering an arbitrary high-frequency resolution^40^. This feature allows obtaining the real and imaginary parts of the dynamic susceptibility at arbitrary frequency components simultaneously, such as 3/2 of the driving frequency as shown in Fig. 1c (see methods for further details). However, when this method is applied to a continuous magnetic layer, spin waves oscillating at 3/2 of the driving frequency are expected, but no corresponding signal is observed by means of SNS-MOKE (see Supplementary Fig. S1). The most likely explanation is that in this case the parametric spin waves are not phase stable during the measurement. To promote phase stability, an elliptical magnetic element is prepared. Here the fixed boundary conditions facilitate the formation of a standing spin-wave mode pattern that is fixed in space. Indeed, above a threshold rf-amplitude, a coherent signal starts to appear (cf. Fig. 1c). Simultaneously, we detect additional coherent non-linear signals at other integer and half-integer multiples as a direct consequence of the strongly anharmonic precession of the magnetization, as demonstrated in Fig. 2. This behavior is a hallmark of the amplitude-phase-oscillations expected for non-linear processes at low bias fields^38^. While the integer multiples^13,16^ cover a field range as broad as the ferromagnetic resonance line shape, the odd half-integer harmonics are restricted to a very narrow field window in which they emerge coherently. Using additional micromagnetic simulations for a comparable sample geometry we find, that the additional half-integer multiples with integer index *n* indeed arise from the precessional motion, spanning a frequency comb *n*⋅f_rf_/2 with amplitudes matching the experimental results, as shown Fig. 2i.

For the non-linear spin-wave process shown in Fig. 2c, one clearly observes a threshold behavior which is in good agreement with the prediction from our analytical k-space model^38^ (see Supplementary Fig. S2). However, as already discussed, the detection of coherent NLSWs at half-integer harmonics strongly depends on the sample geometry. Thus, the values obtained by SNS-MOKE might be larger compared to the simulations due to the lack of phase stability near the threshold. While one can observe phase-stable NLSW generation in larger elliptical elements, even smaller structures, such as a 5 μm × 4 μm rectangle, shows no phase-stable NLSW response in our measurements. We attribute this effect to rapid thermal fluctuations of the phase of the non-linear response.

### Phase stability

**Fig. 3:**
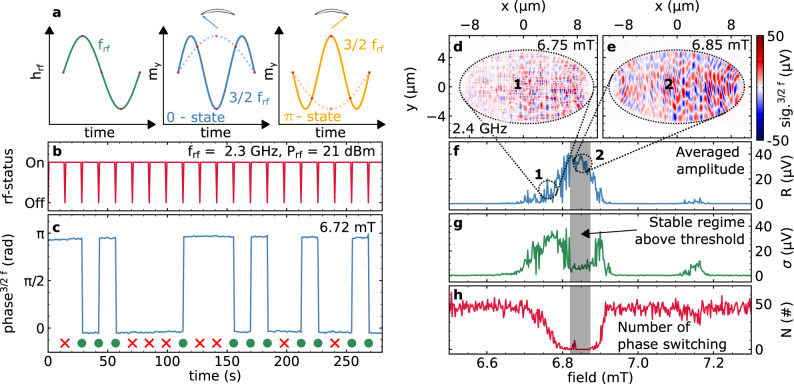
Phase stability of NLSWs. **a** The rf-driving field at f_rf_ (green) results in parametrically generated magnons at half-integer multiples of the driving frequency with two distinct phase states. The solid lines depict the 0-state in blue and the *π*-state in yellow for the 3/2 f_rf_ non-linearity while the dotted line show the corresponding states for 1/2 f_rf_. **b** The rf-power modulation as a function of time while the phase of the NLSW response is depicted in **c**. The phase of the NLSW signal randomly jumps to the *π*-state or stays in the 0-state after switching the rf-output back to the initial level. Switching events are depicted by green dots. Panels **d** and **e** show two spatially-resolved measurements of a 20 μm × 10 μm elliptical element at slightly different field values revealing different regions of stability. **f**, **g**, and **h** Field-dependent measurements performed in the center of the ellipse where the signal is detected for 50 s at each field step resulting in an averaged amplitude *R*, a corresponding standard deviation *σ* and a counter for the number of phase switching events N, respectively.

The fact that a direct observation of NLSWs is only possible within a narrow field window and in certain element geometries requires further attention. Especially since indirect evidence for the onset of these non-linearities is observed for all investigated structures and in agreement with micromagnetic simulations. For conventional parametric spin-wave processes it is known that the resulting non-linear magnon pair populates one of two energetically equivalent phase states either in-phase or out-of-phase with respect to the rf-excitation^42^. In the case of three-magnon scattering in parallel pumping geometry randomly occurring phase transitions between the 0-state and the *π*-state appear stochastically with equal probability. In our experiments, we identify such degenerate phase states for the transverse pumping geometry, as depicted in Fig. 3a and reveal their stochastic switching behavior in phase-resolved measurement of the 3/2 f_rf_ non-linearity as randomly emerging sign reversals of the signal. Note that for SNS-MOKE experiments phase stability of the excited NLSWs with respect to the excitation on time scales larger than the measurement bandwidth is essential to obtain a coherent signal. For this purpose, we investigate how experimental parameters such as the excitation amplitude and the bias field influence phase stability.

Interestingly, near the threshold condition, the NLSW signals appear to be less stable in phase over time, resulting in randomly occurring phase flips between the two distinct states most likely caused by thermal fluctuations. A further increase of the rf-driving power stabilizes the NLSWs over a broader field range allowing for phase-stable spatially-resolved imaging, as demonstrated in Fig. 3e. However, even in this stable regime, the initial phase of the NLSWs with respect to the excitation is set randomly to one of the degenerate states. This is demonstrated in Fig. 3b, c where the NLSW is prepared in the stable regime while a brief interruption of the rf-power results either in a 180° phase flip or leaves the phase unchanged with equal probability. This observation illustrates the existence of two degenerate phase states from which one is randomly chosen as soon the non-linearity at 3/2 f_rf_ sets in. In fact, whenever half-integer harmonics of the driving signal are generated a twofold degeneracy of the possible phase state occurs (cf. Fig. 3a). Thus, in contrast to SNS-MOKE, conventional TR-MOKE experiments with rf-amplitude or phase modulation would be unable to detect such NLSW signals due to a periodic resetting to a random phase state. In the next step, we aim for a better understanding of the stability criteria of the NLSWs. Therefore, we slowly sweep the external magnetic field and detect the phase stability for each step on the time scale of 50 s (cf. Fig. 3f–h). Here, we reveal a regime of enhanced stability where phase flips are less likely (gray area). For slightly larger (or smaller) bias fields the stability is reduced as indicated by the occurrence of switching events on the time-scale of a few seconds, as recently reported for three-magnon scattering processes^42^. In Fig. 3f–h we demonstrate how the increasing number of switching events in the vicinity of the stable regime results in a decreased average amplitude and an enhanced standard deviation of the measured signal. In comparison to this small field range of enhanced stability micromagnetic simulations indicate that the field range in which 3/2 f_rf_ non-linear spin waves are generated is as broad as the line width of the ferromagnetic resonance (see Supplementary Fig. S3). Moreover, the formation of this stable regime, where phase flips are mostly prevented, strongly depends on the chosen element geometry. In SNS-MOKE experiments, therefore only a small fraction of the entire NLSW spectrum is observable as random phase flips due to thermal fluctuations occur on a time scales faster than the measurement bandwidth, resulting in a loss of signal.

### Spin-wave imaging

**Fig. 4:**
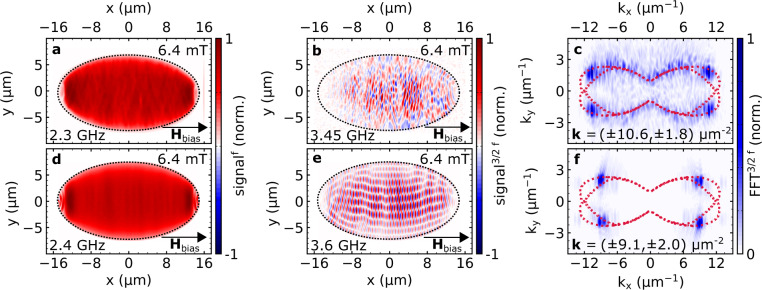
Spatially-resolved imaging of NLSWs and comparison to simulations. Spatially-resolved SNS-MOKE measurements analyzed at f_rf_ and 3/2 f_rf_ are shown for an elliptical element (30 μm × 15 μm) in **a** and **b**, respectively. While **a** shows a uniform signal across the entire structure the 3/2 f_rf_ response in **b** appears completely different and consists of a complex spin-wave pattern. By utilizing a 2D-FFT the wave vector components of this standing spin-wave pattern can be accessed as presented in **c**. Here, the dotted red lines, depict the iso-frequency line from non-linear spin-wave dispersion obtained with the analytical model. In **d** and **e**, micromagnetic simulations for the same element geometry are presented using similar conditions. The resulting 2D-FFT is depicted in **f** indicating the same spin-wave pattern with slightly smaller wave vector components.

The experimental observation of phase-stable NLSWs at 3/2 f_rf_ implies that the predicted amplitude-phase-oscillations^38^ occur coherently with respect to the driving frequency in a certain bias field window. In the phase-stable regime (cf. Fig. 3), SNS-MOKE allows for spatially-resolved imaging of the NLSWs and directly reveals the in-plane wave vector components of the parametrically generated magnons at 3/2 f_rf_. For this type of measurement, all experimental parameters are fixed while real and imaginary parts of the polar magnetic response are recorded in a point-wise fashion. Here, a mostly uniform signal is obtained at the driving frequency f_rf_ = 2.3 GHz, as shown in Fig. 4a, while above the threshold condition a phase-stable pattern of NLSWs is detected, oscillating at a frequency of 3/2 f_rf_ = 3.45 GHz (see Fig. 4b). At the same time, additional non-linear signal components due to the amplitude-phase-oscillations are detected at other half-integer multiples, as demonstrated in Supplementary Fig. S4. Corresponding micromagnetic simulations of the non-linear magnetization dynamics are carried out and compared to the experimental results, as shown in Fig. 4d, e, respectively. The direct comparison of the excited wave vectors in the standing spin-wave patterns is achieved by performing a two-dimensional fast Fourier transform (2D-FFT) for experimental data and the simulation. For both cases, the 2D-FFT pattern shows the predicted NLSW pairs at 3/2 f_rf_^38^. Furthermore, we find a good agreement between our results and the analytical model as we compare the obtained non-linear wave vector components from spatially-resolved imaging with our calculations as a function of the rf-frequency (see Supplementary Fig. S5). The non-linear effects we observe convert the uniform mode into short-wavelength dipole-exchange spin waves within the ferromagnetic material without the need for patterning while the wavelength can be tuned by external parameters. This is an advantage compared to recent concepts of generating short-wavelength spin waves^19,20^ using periodic metallic coupling structures.

We would like to point out that the observed spin-wave patterns emerge in a frequency-locked regime^38^, additional signal components are visible and follow the iso-frequency line of the non-linear dispersion at 3/2 f_rf_ obtained from the analytical model (red line in Fig. 4). We explain this population of different modes by elastic magnon–magnon scattering processes facilitated by slight spatial variations of magnetic properties as it can be expected for a polycrystalline Ni_80_Fe_20_ sample at low bias fields. To support this assumption, the impact of spatially varying magnetic properties is demonstrated using micromagnetic simulations, as shown in Supplementary Fig. S6. In the following, we aim to achieve phase control by stabilizing the NLSWs in one of the degenerate phase states.

### Phase-locking of non-linear spin waves

**Fig. 5:**
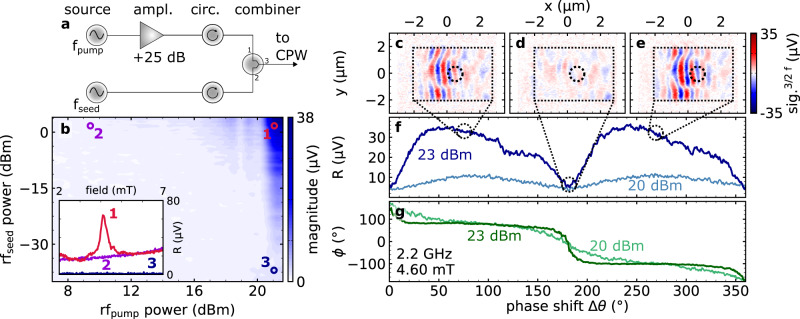
Phase-locking of NLSWs. **a** Modified excitation scheme for phase-locking experiments with additional seed frequency. **b** NLSW response as a function of pump and seed frequency power levels for a 5 μm × 4 μm Ni_80_Fe_20_ element. The inset shows three different regimes of power level combinations: (1) A signal trace at a large seed power level with a large pump power which contains NLSWs in red, (2) the off-resonant background due to direct excitations at 3/2 f_rf_ with low pump power in violet, and (3) low seed power and large pump power in blue. We subtract the signal of the direct excitation for each seed power level to reach the regime of seeded NLSW generation in **b**. In **c**–**e** spatially-resolved images of the spin-wave signals analyzed at 3/2 f_rf_ are shown for different phase shifts Δ*θ* between f_rf_ and 3/2 f_rf_ sources. In **f**, a more detailed view on the magnitude R is presented where the black markers indicate the situations depicted in **c**–**e**. Panel **g** shows the signal's phase *ϕ* obtained in the center of the element as a function of phase shift Δ*θ*.

The integration of NLSWs into magnon-based devices requires direct control of propagation direction, amplitude or phase. Furthermore, the synchronization of excited non-linear magnons to an external reference frequency has enormous potential for magnon-based computing^23,25–27^. Phase control in such a non-linear system would allow to precisely set the NLSW’s initial phase and prevent the phase state from random switching events by increasing its robustness against thermal fluctuations. For this, we modify the excitation scheme by integrating a second rf-source^43^ operating at 3/2 f_rf_, as shown in Fig. 5a. This allows to seed the magnetic system with an additional frequency component at the desired phase. Physically, phase-locking is achieved due to the coupling between the homogeneous rf-field and the NLSWs for the following reasons: (i) As NLSWs are generated within a confined element their standing spin-wave pattern is fixed in space even if the phase state randomly switches. As a result of these boundary conditions only two degenerate phase states exist. (ii) The interplay of a uniform rf-field with the boundaries of the elements results in non-uniform dynamic demagnetization fields^44^. Here, the non-uniformity of the internal fields provides a spectrum of wave vectors in the range of the NLSWs with an relative amplitude of a few percent with respect to the uniform contribution. The presence of this rf-field breaks the symmetry of the initially degenerate states and allows to select only one of them. As soon as coherent magnons with the corresponding wave vectors are provided by the seed rf-field, the NLSWs will parametrically amplify this specific mode with the phase of the seed source.

The applied power level of the seed frequency component is typically two orders of magnitude smaller compared to the pump excitation. This modified excitation scheme is now applied to one of the elements which do not show a coherent NLSW signal without a seed excitation, namely a 5 μm × 4 μm rectangular element (cf. Supplementary Fig. S1). In the following, we analyze the signal amplitude at 3/2 f_rf_ while the power level of rf-pump and rf-seed were varied in a step-wise fashion. Figure 5b shows the obtained 2D map. In agreement with our analysis above, a regime exists where the coherent excitation of NLSWs only occurs when an additional seed amplitude (at f_seed_ = 3/2 f_rf_) is applied. We would like to point out, that due to the amplitude-phase-oscillations, the introduced stabilization process can also be performed at other odd half-integer seed frequencies, as demonstrated in Supplementary Fig. S10. Here, the separation of seed and detection frequency reduces the influence of direct excitations at the detection frequency, and thus provides a pure NLSW signal. Clearly, the seed frequency sets and stabilizes the phase of the obtained NLSW signal.

Next, we study the phase control of the excited modes for the 5 μm × 4 μm rectangular magnetic element. In a discrete structure, one can expect that only two phase states with respect to the excitation are possible for a standing spin wave. For this purpose spatially-resolved imaging is performed with both power levels set to the stable regime above threshold, while the phase of the seed (at 3/2 f_rf_) was varied with respect to the phase of 3/2 of the pump frequency for the images shown in Fig. 5c–e. Here, the amplitude of the NLSW signal is strongly suppressed for a relative phase Δ*θ* = 180° (cf. Fig. 5d), while the sign of the signal changes from c to e corresponding to the two distinct phase states. Measuring the magnitude of the signal in Fig. 5f as a function of Δ*θ* in the center of this device results in a 180° periodicity. At the same time the phase of the signal *ϕ* shifts by 180°, as shown in g. We link the rapid phase jump in g to the transition from one of the two possible phase states to the other while the magnitude returns to the same level as the phase of the seed frequency is varied. For the transition region the two phase states are equally probable and the signal vanishes. The observation of these two possible phase states and their accessibility via phase shifts of the seed frequency is further corroborated by micromagnetic simulations (cf. Fig. S11). Note that the observed phase-locking effect strongly depends on the applied pump (f_rf_) and seed (3/2 f_rf_) power levels. For example, as the pump power is slightly decreased, the transition between the states becomes less sharp and at the same time, the magnitude is reduced. However, the applied scheme allows to initialize the NLSW in a defined and stable phase state, and provides a direct control for setting the phase state.

## Discussion

In the experiments, we found that a series of signals at half-integer multiples of the driving frequency arises and their spatial distribution can be imaged under phase-stable conditions. The agreement between the experimental data and k-space modeling as well as micromagnetic simulations is excellent. Moreover, by utilizing the phase sensitivity of our imaging technique we revealed different regimes of stability. Within the stable regime, we found that the excitation tends to stochastically populate one of two degenerate phase states. As we demonstrated, these phase states of the NLSWs can be easily manipulated by external rf-fields illustrating their potential for phase-encoded information processing. A possible application of our results is that externally stabilized non-linear spin waves are integrated into magnon-based devices, e.g., as phase-locked large wave vector spin-wave emitters. We point out that the required very low bias fields may be generated using electrical currents flowing in microscopic wire structures. This should also enable magnonic devices, such as spin-wave amplifiers that are crucial for realizing magnonic computing applications.

## Methods

### Sample preparation

The metallic structures were prepared by electron beam lithography, thermal evaporation and lift-off processes on top of gallium-arsenide (GaAs) substrates. First, a 100 nm thick gold CPW was deposited. The 50 Ω CPW has a gap width of 25 μm while the signal line is 50 μm wide. On top of the signal line of the CPW 20 nm thick Ni_80_Fe_20_ elements were structured by electron beam lithography and lift-off processes. The sample geometry provides mostly uniform in-plane excitation in the center of the waveguide where the elements are located. Various different geometries were prepared on the sample: Elliptical and rectangular elements with lateral dimensions of 40 μm × 20 μm, 30 μm × 15 μm and 20 μm × 10 μm, 5 μm × 4 μm, respectively. A second sample, used for initial measurements shown in Supplementary Fig. S1, was prepared in a similar fashion but here the entire signal line of the CPW is covered with a 20 nm thick Ni_80_Fe_20_ film.

### SNS-MOKE

In the presented experiments, a 520 nm laser with a pulse length of 200 fs is focused with a high numerical aperture objective lens onto the sample. The dynamics within the Ni_80_Fe_20_ elements are excited by the uniform in-plane rf-magnetic field generated by the CPW. In SNS-MOKE^40^ measurements the femtosecond laser is utilized to sample the magnetization dynamics in a phase-resolved fashion. Phase-stable measurements become possible when laser, rf-source, and lock-in amplifier (used for the detection) are stabilized to the same master clock. The train of ultrafast laser pulses with a repetition rate f_rep_ = 80 MHz corresponds to a frequency comb with a spacing f_rep_ of the comb lines and converts the GHz precessional motion of the spins down to the intermediate frequency *ε* derived from the offset of the rf-frequency f_rf_ to the nearest laser comb line *n*⋅f_rep_. This process leads to an inherent modulation of the dynamics and allows to detect the precessional motion without the loss of phase or amplitude information at kHz or MHz alias frequencies by direct demodulation at this frequency component *ε*. This technique allows for simultaneous detection of the real and imaginary part of the dynamic susceptibility due to the polar magneto-optical Kerr effect. The obtained signal components allow to reconstruct the magnitude and phase of the *M*_**z**_ component for arbitrary detection frequencies. As an example one may use an excitation frequency of f_rf_ = 2.001 GHz, generating the lowest alias frequency at *ε* = ∣f_rf_ − 25⋅f_rep_∣ = 1 MHz. For the detection of a signal component at 3/2 f_rf_ = 3.0015 GHz, the signal needs to be demodulated at *ε*_non-linear_ = 1.5 MHz. For simultaneous measurements at different frequencies, a multi-frequency lock-in amplifier is used. The rf-pumping signal is amplified up to 23 dBm using a broadband rf-amplifier to access the non-linear regime. For the phase locking experiments a power combiner is used to join the amplified f_rf_ component and the seed frequency component. Both frequency sources are are mutually isolated by rf-circulators (see Fig. 5a).

### Analytical model

To describe the observed phenomena, we utilize a model introduced by Bauer et al.^38^ to obtain the spin-wave life times and the non-linear dispersion in k-space within the limit of strong modulation. Therefore, the LLG equation is transformed into the Mathieu equation by substituting the magnetization component with *f* as described in ref. 38. This results in

1 $$f^{\prime\prime}+(a-2q\cos (2x))f=0\,,$$

where, $a={({\omega }_{{{{{{{{\rm{k}}}}}}}}}/{\omega }_{{{{{{{{\rm{mod}}}}}}}}})}^{2}$ includes the spin-wave frequency *ω*_k_ as well as the modulation frequency ${\omega }_{{{{{{{{\rm{mod}}}}}}}}}$, while *q* depicts the modulation strength. The Mathieu equation can be solved by the ansatz

2 $$F(a,q,x)={e}^{i\nu (a,q)x}\cdot P(a,q,x)\,,$$

where *ν*(*a*, *q*) is the complex Mathieu exponent and *P* denotes a periodic function. The real part of *ν*(*a*, *q*) results in the (non-linear) spin-wave dispersion as a function of *k*_x_ and *k*_y_. The imaginary part yields the spin-wave life time and allows to determine threshold values when non-linear spin waves become critical as a function of the rf-driving field amplitude for fixed rf-frequency and bias field. For this threshold condition, the wave-vector components of the 3/2 f_rf_ spin waves can be accessed in a frequency-resolved manner. For the calculation, we used a Ni_80_Fe_20_ thickness of 20 nm, the saturation magnetization *M*_s_ = 800 kA/m, the exchange constant *A*_exch_ = 13 pJ/m, gyromagnetic ratio γ = 1.76 × 10^11^(T^−1^s^−1^), and a damping constant of *α* = 0.008.

### Micromagnetic simulations

The micromagnetic simulations complementing our experimental findings, are performed using the GPU-accelerated software package mumax3^45^. The time- and space-dependent effective magnetic field is defined by the functional derivative of the free energy density ${{{{{{{\mathcal{F}}}}}}}}[{{{{{{{\bf{m}}}}}}}}]$ with respect to the unit vector field of the magnetization **m**(**r**, *t*)

3 $${{{{{{{{\bf{B}}}}}}}}}_{i}^{{{{{{{{\rm{eff}}}}}}}}}(t)={{{{{{{{\bf{B}}}}}}}}}_{i}^{{{{{{{{\rm{ext}}}}}}}}}(t)+{{{{{{{{\bf{B}}}}}}}}}_{i}^{{{{{{{{\rm{exch}}}}}}}}}+{{{{{{{{\bf{B}}}}}}}}}_{i}^{{{{{{{{\rm{d}}}}}}}}}+{{{{{{{{\bf{B}}}}}}}}}_{i}^{{{{{{{{\rm{a}}}}}}}}}\,.$$

It is composed of the external field ${{{{{{{{\bf{B}}}}}}}}}_{i}^{{{{{{{{\rm{ext}}}}}}}}}(t)$, including the static bias field and the oscillating excitation field contribution; the exchange interaction field ${{{{{{{{\bf{B}}}}}}}}}_{i}^{{{{{{{{\rm{exch}}}}}}}}}=(2{A}_{{{{{{{{\rm{exch}}}}}}}}}/{M}_{{{{{{{{\rm{s}}}}}}}}}){{\Delta }}{{{{{{{{\bf{m}}}}}}}}}_{i}$, with the exchange stiffness *A*_exch_ and the saturation magnetization *M*_s_, the demagnetizing field ${{{{{{{{\bf{B}}}}}}}}}_{i}^{{{{{{{{\rm{d}}}}}}}}}={M}_{{{{{{{{\rm{s}}}}}}}}}{\hat{{{{{{{{\bf{K}}}}}}}}}}_{ij}*{{{{{{{{\bf{m}}}}}}}}}_{j}$, where we refer to ref. 45 for details of the calculation of the demagnetizing kernel $\hat{{{{{{{{\bf{K}}}}}}}}}$. The magnetization dynamics is described by the Landau Lifshitz Gilbert equation (LLG). The effective magnetic field enters the LLG equation as

4 $${\dot{{{{{{{{\bf{m}}}}}}}}}}_{i}(t)=-\frac{\gamma }{1+{\alpha }^{2}}\left[{{{{{{{{\bf{m}}}}}}}}}_{i}\times {{{{{{{{\bf{B}}}}}}}}}_{i}^{{{{{{{{\rm{eff}}}}}}}}}+\alpha {{{{{{{{\bf{m}}}}}}}}}_{i}\times \left({{{{{{{{\bf{m}}}}}}}}}_{i}\times {{{{{{{{\bf{B}}}}}}}}}_{i}^{{{{{{{{\rm{eff}}}}}}}}}\right)\right]\,.$$

This equation is solved for each simulation cell *i* of the discretized magnetization vector field **m**_*i*_. In the simulations we used the same material parameters as in the analytical modelling.

## Supplementary information

Supplementary information

## Data Availability

All primary data that support our findings of this study, as well as the code employed in simulations, are available at Zenodo^46^.
