# Supplementary material for: Imaging and phase-locking of non-linear spin waves
Source: Nat Commun. 2022 Aug 23;13:4939. doi: 10.1038/s41467-022-32224-0 (PMC9399154; doi:10.1038/s41467-022-32224-0)
Supplement: Supplementary file 1 — Supplementary information [file 41467_2022_32224_MOESM1_ESM.pdf]

Supplementary information for: Imaging and phase-locking of non-linear spin waves

Rouven Dreyer[1], Alexander F. Schäffer[1], Hans G. Bauer[2], Niklas Liebing[1], Jamal Berakdar[1], and Georg Woltersdorf[1,3,*]

[1]Institute of Physics, Martin Luther University Halle-Wittenberg, Von-Danckelmann-Platz 3, 06120 Halle, Germany
[2]Jahnstrasse 23, 96050 Bamberg, Germany
[3]Max Planck Institute of Microstructure Physics, Weinberg 2, 06120 Halle, Germany
[*]Corresponding author: Georg Woltersdorf, georg.woltersdorf@physik.uni-halle.de

July 8, 2022

This PDF includes:

1. Supplementary Figures:

S1. Response at f_{rf} and $3/2\ f_{\mathrm{rf}}$ for different sample geometries.
S2. Threshold analysis of $3/2\ f_{\mathrm{rf}}$ NLSWs.
S3. Micromagnetic simulations of the field resolved spin-wave spectra analyzed at different frequencies with different spatial variations of the saturation magnetization.
S4. Experimentally obtained signal amplitudes at different frequency components.
S5. Wave vector analysis as a function of the rf-frequency.
S6. Micromagnetic simulations of spatially-resolved NLSW excitations with different spatial variations of the saturation magnetization.
S7. Influence of the in-plane field orientation on NLSW generation.
S8. NLSW pattern in $5\,\mu\mathrm{m} \times 4\,\mu\mathrm{m}$ element with seed frequency.
S9. Micromagnetic simulation of non-linear spin-wave generation in different element geometries.
S10. NLSW signal obtained at different seed frequencies.
S11. Micromagnetic simulation of the phase-locking of NLSWs to an external seed frequency.

Supplementary Figures

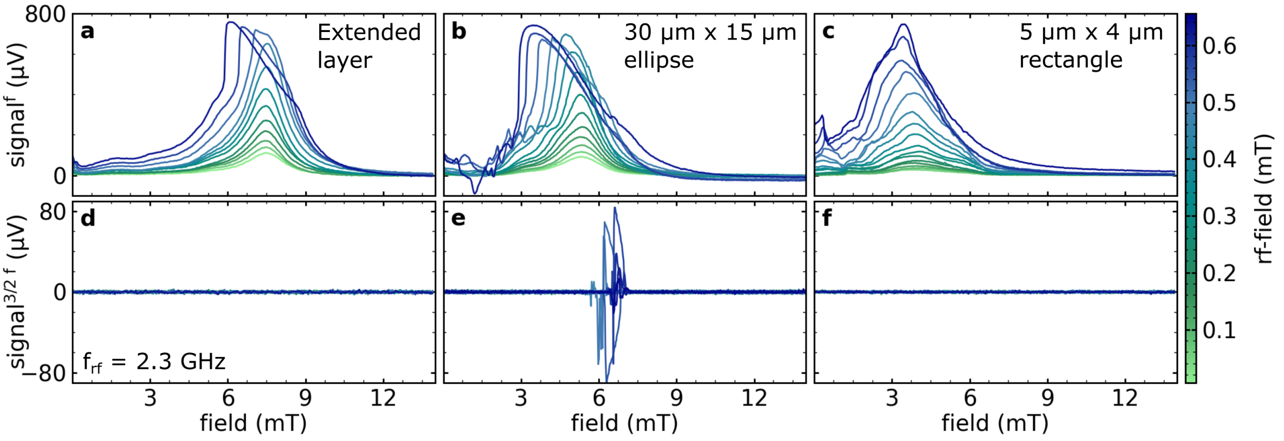

Fig. S1 | Response at f_{rf} and $3/2\ f_{rf}$ for different sample geometries. We performed field swept measurement with increasing rf-power levels in different sample geometries to identify suitable conditions for coherent NLSW generation. Panels **a**, **b** and **c** show the ferromagnetic resonance spectra for an extended layer of $20\,\mathrm{nm}$ thick $Ni_{80}Fe_{20}$, a $30\,\mu\mathrm{m} \times 15\,\mu\mathrm{m}$ ellipse and a $5\,\mu\mathrm{m} \times 4\,\mu\mathrm{m}$ rectangular element, respectively. All measurements indicate the onset of non-linear processes by showing a phase shift and a saturation of the uniform mode. However, only in panel **e** a phase coherent non-linear response is detectable at $3/2\ f_{rf}$ for the $30\,\mu\mathrm{m} \times 15\,\mu\mathrm{m}$ ellipse, while the other two geometries show no phase-stable NLSWs as shown in **d** and **f**. Moreover, we were able to obtain NLSW generation in all rectangular and elliptical structures with dimensions larger than $20\,\mu\mathrm{m} \times 10\,\mu\mathrm{m}$.

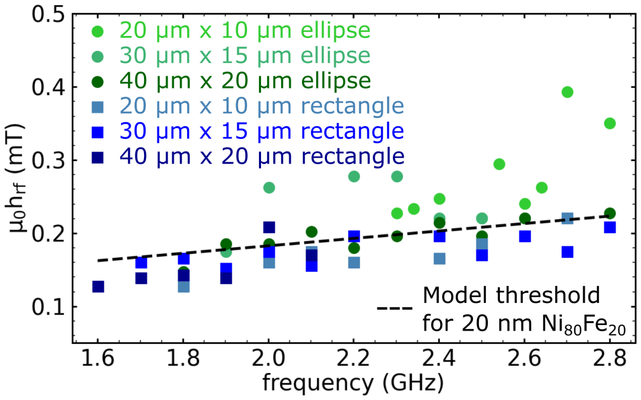

Fig. S2 | Threshold analysis of $3/2\ f_{rf}$ NLSWs. We performed power-dependent measurements at different element geometries on the same sample and extracted the threshold rf-field as a function of the driving frequency. The blue values (squares) are obtained on rectangular elements while the green data points (circles) correspond to elliptical shaped $Ni_{80}Fe_{20}$ elements. The dashed black line indicates the threshold values obtained with the analytical model for a $Ni_{80}Fe_{20}$ thickness of $20\,\mathrm{nm}$.

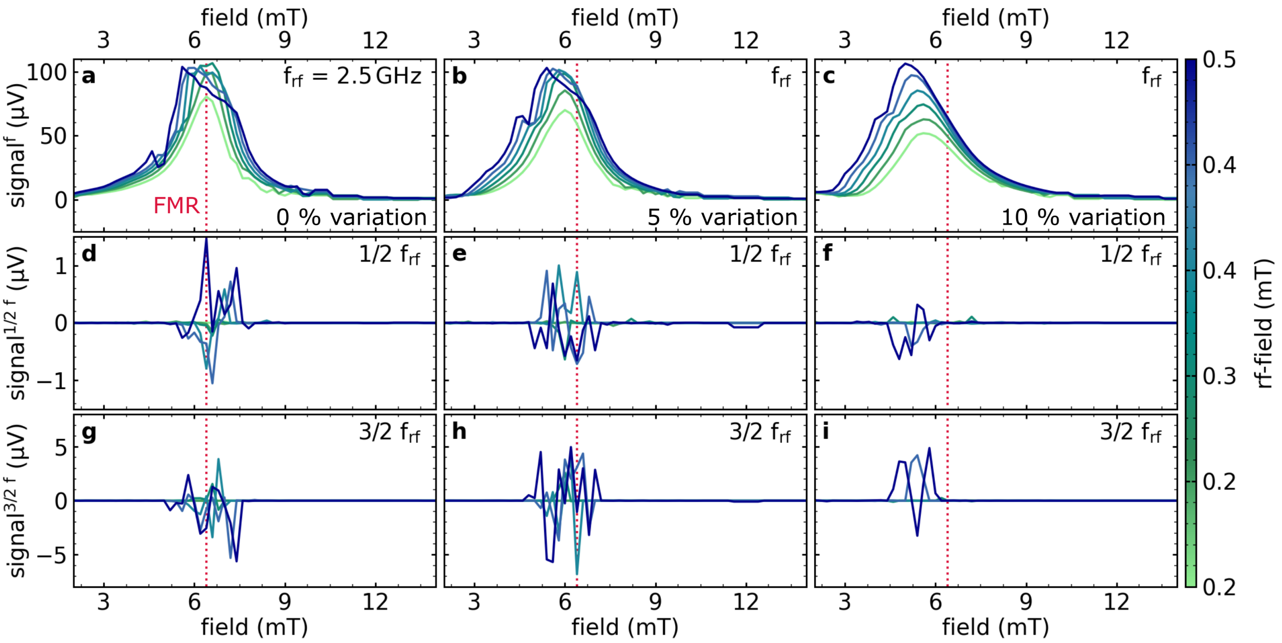

Fig. S3 | Micromagnetic simulations of the field resolved spin-wave spectra analyzed at different frequencies with different spatial variations of the saturation magnetization. Rf-power dependent simulations of the field-resolved spin-wave spectra. (**a**)-(**c**) The imaginary part of the response is analyzed at the driving frequency which results in typical Lorentzian FMR line shapes for low-power driving amplitudes. For increasing rf-power levels the phase starts to shift and the mode saturates. From panel **a** to **c** the spatial variation of M_s was increased from 0 % up to 10 % (the size of the grains is $100\,\mathrm{nm}$). For larger variations of M_s the FMR condition is shifted towards lower fields, as depicted in **b** and **c** (the dotted red line shows the low power FMR condition for 0 % variation). Panel **d-e** show the imaginary part of the $1/2$ f_rf response while **g-i** display the $3/2$ f_rf components. Note that the field range in which NLSW generation appears is broad. The experimentally obtained coherent part of the spectrum is on the order of $1\,\mathrm{mT}$, while the incoherent part accessible by the simulation covers a field range of $3\,\mathrm{mT}$ (corresponding to the line width of the ferromagnetic resonance) with a symmetric distribution around the FMR condition.

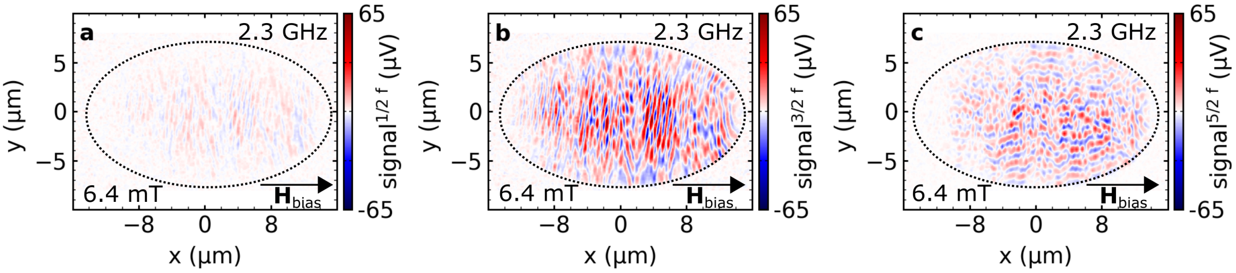

Fig. S4 | Experimentally obtained signal amplitudes at different frequency components. We compare the different signal amplitudes obtained at $1/2$ f_rf, $3/2$ f_rf and $5/2$ f_rf shown in **a**, **b** and **c**, respectively. The signal ratio taken from such measurements between $3/2$ and $1/2$ f_rf is on the order of 6:1, while the ratio from $3/2$ and $5/2$ is on the order of 3:1. The wave vector components in the $1/2$ and $3/2$ f_rf channel appear to be similar, while the $5/2$ f_rf signal contains higher-order wave vector components.

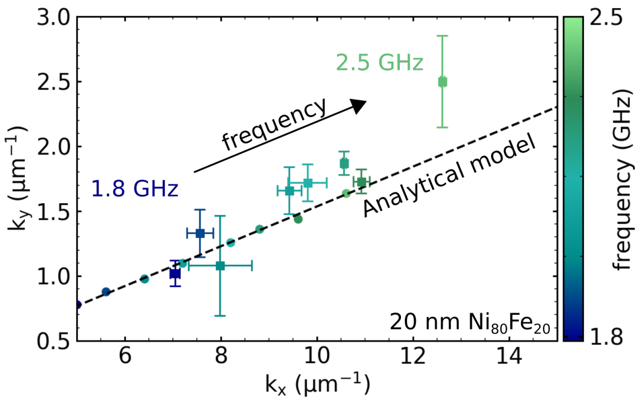

Fig. S5 | Wave vector analysis as a function of the rf-frequency. By extracting the wave vectors of the NLSWs oscillating at $3/2$ f_{rf} by means of 2D-FFT, we directly access the k_x and k_y component of the NLSWs. We performed such measurements at various frequencies and compare the obtained values with the results of the analytical model (black dashed line as guide for the eye). The experimental results shown as squares and their error bars are color coded indicating the different frequencies, the same color code is used for the points in the figure obtained from the model showing slightly smaller wave vector components.

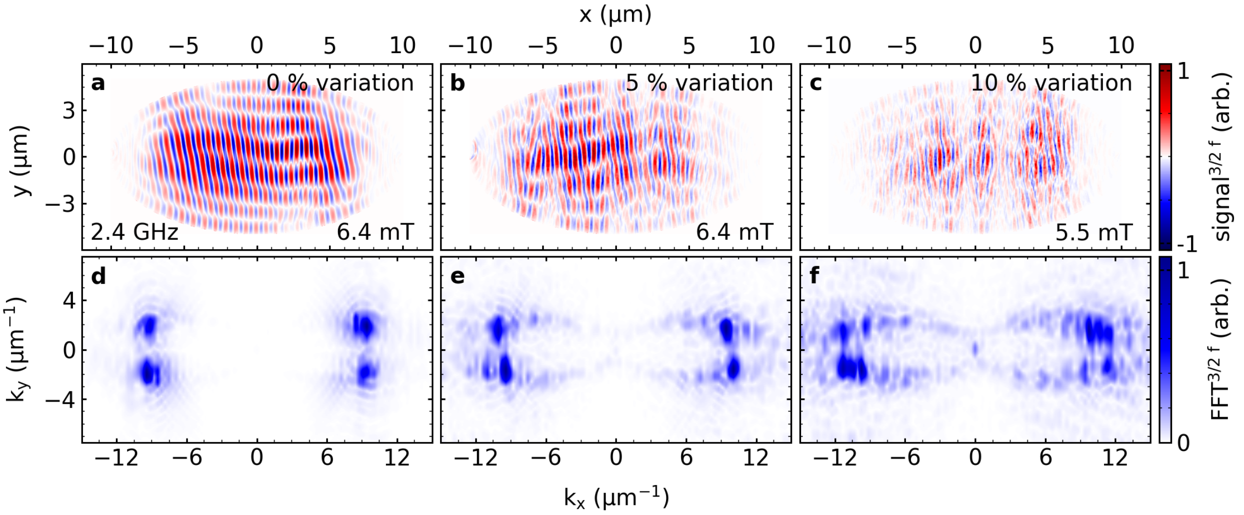

Fig. S6 | Micromagnetic simulations of spatially-resolved NLSW excitations with different spatial variations of the saturation magnetization. Real space images of NLSWs oscillating at $3/2$ f_{rf} obtained from micromagnetic simulations of a $20\,\mu m$ x $10\,\mu m$ ellipse. From panel **a** to **c** the spatial variation of M_s was increased from 0 % up to 10 % (the size of the grains is $100\,nm$). In **d-e** the corresponding 2D-FFTs are shown. By increasing the strength of the variation additional magnon-magnon scattering events occur resulting in the population of the magnon modes on the iso-frequency lines. The rf-power level in all images is above the threshold condition.

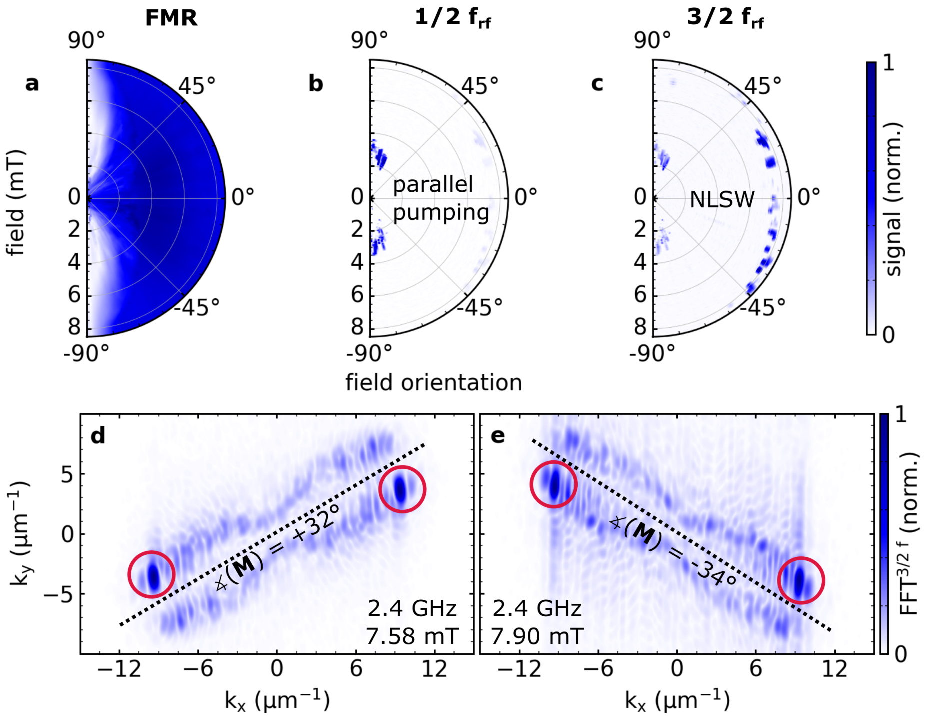

Fig. S7 | Influence of the in-plane field orientation on NLSW generation. (**a**)-(**c**) The response of the system is detected as a function of the external bias field for different in-plane field orientations at f_{rf}, $1/2\ f_{rf}$ and $3/2\ f_{rf}$, respectively. At $\pm 90°$ the uniform response in **a** is strongly suppressed while three-magnon scattering effects give rise to signal components at $1/2\ f_{rf}$ in **b**. This corresponds to the parallel pumping geometry. In the range between $\pm 45°$ the dominant non-linear effect becomes visible at $3/2\ f_{rf}$ in **c**. The obtained spin-wave pattern from spatially-resolved imaging at $3/2\ f_{rf}$ for different angular orientations is demonstrated in **d** and **e**. In **d** the external field's direction is changed by $32°$ resulting in a rotation of the NLSW pattern obtained by performing a 2D-FFT. A change of $-34°$ leads to the pattern shown in **b**. As indicated by red circles, the symmetry of the pattern obtained for $0°$ orientation changes and the spin waves tend to align along the direction with smaller k_y components.

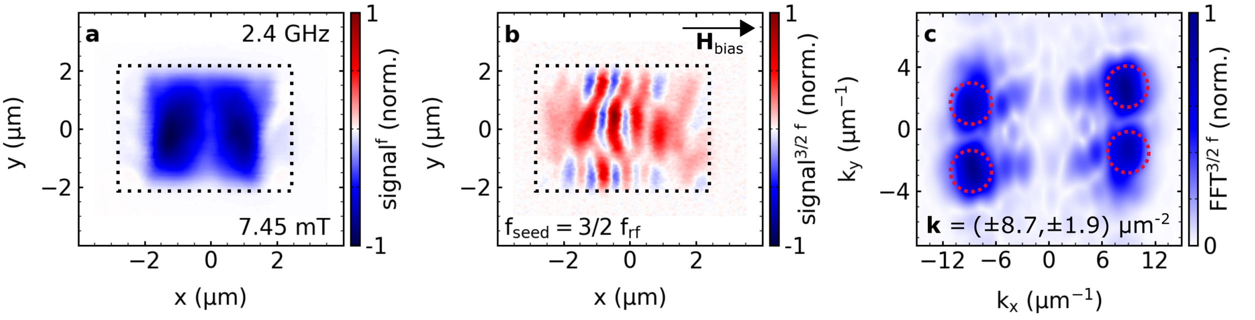

Fig. S8 | NLSW pattern in 5 µm × 4 µm element with seed frequency. SNS-MOKE measurements analyzed at f_{rf} = 2.4 GHz and 3/2 f_{rf} were shown for an rectangular element (5 µm × 4 µm) in **a** and **b**, respectively. While panel **a** shows a mostly uniform signal across the entire structure the 3/2 f_{rf} signal in **b** consists of a complex spin-wave pattern superimposed with a more uniform background due to the direct excitation at this frequency. In this element, the obtained NLSW pattern only becomes accessible due to the applied seed frequency component. By utilizing a 2D-FFT the wave vector components of this pattern can be accessed as presented in **c**. Note that for a better visibility of the NLSW we subtracted the direct excitation background.

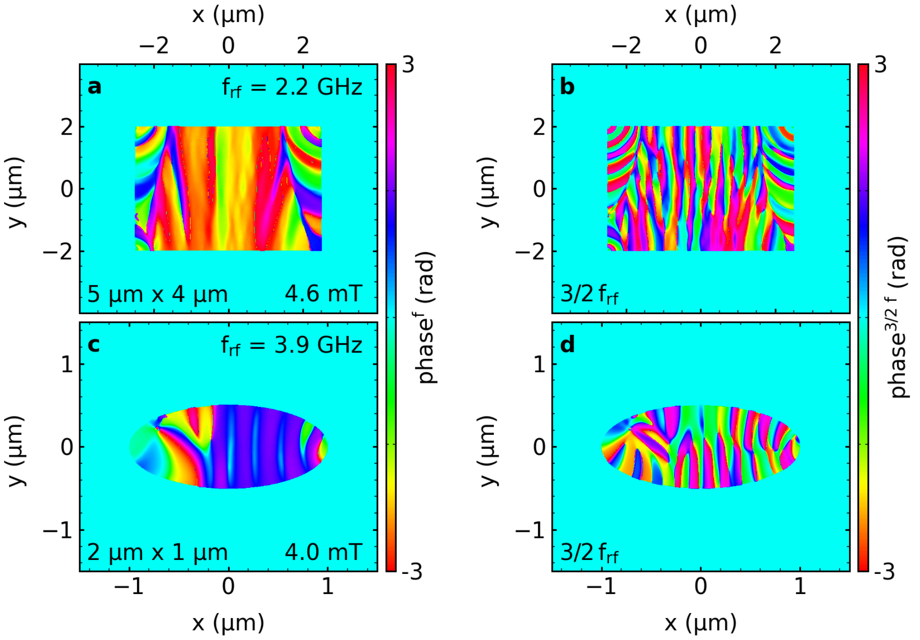

Fig. S9 | Micromagnetic simulation of non-linear spin-wave generation in different element geometries. We performed micromagnetic simulations for a 5 µm × 4 µm rectangular and a 2 µm × 1 µm elliptical shaped element with magnetic properties of $Ni_{80}Fe_{20}$ as shown in **a** and **c**, respectively. We analyzed the response of the system at f_{rf} and 3/2 f_{rf}. Above threshold condition a non-linear spin-wave pattern with the expected wave vector components becomes visible. Note that the resonance condition for small elements shifts significantly to larger frequencies.

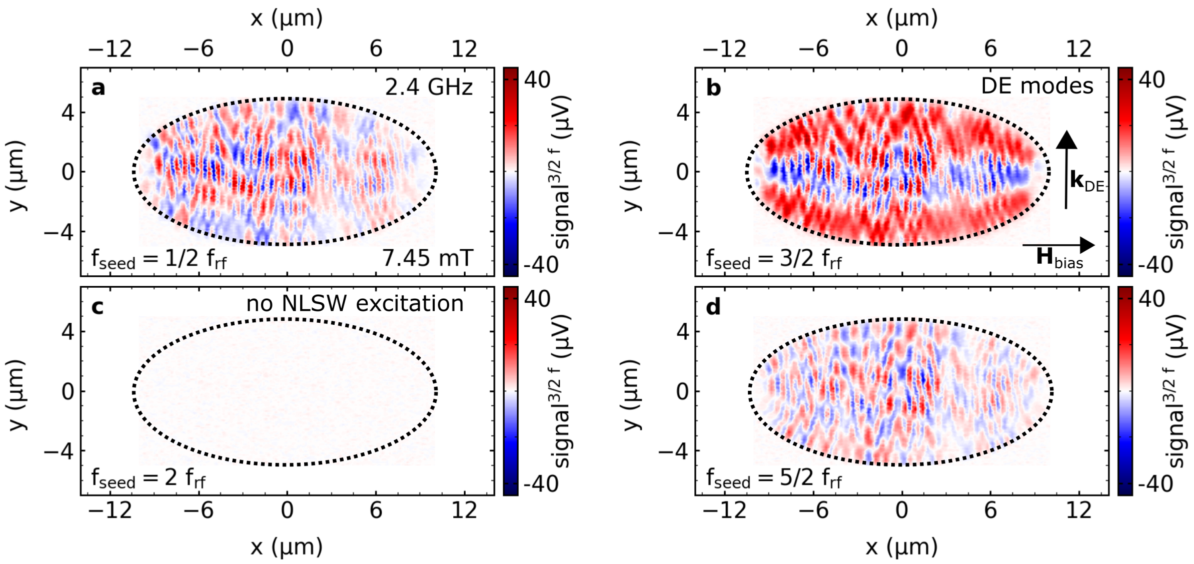

Fig. S10 | NLSW signal obtained with different seed frequencies. The spatially-resolved NLSW signals oscillating at $3/2\ \mathrm{f_{rf}}$ is recorded with different seed frequencies in the $20\,\mu\mathrm{m} \times 10\,\mu\mathrm{m}$ ellipse. In panel **a** and **d** half-integer seed frequencies of $1/2\ \mathrm{f_{rf}}$ and $5/2\ \mathrm{f_{rf}}$ were used, respectively. These seed components result in a purely non-linear signal in the read-out channel since the detected NLSW signal at $3/2\ \mathrm{f_{rf}}$ contains no additional background due to direct excitations as shown in **b** where a $3/2\ \mathrm{f_{rf}}$ seed was used. In order to illustrate, that half-integer seed frequency signals are required, we show as an example $\mathrm{f_{seed}} = 2\ \mathrm{f_{rf}}$ in panel **c**. Here, no phase stability of the NLSW excitation is obtained. The seed power levels were set to $0\,\mathrm{dBm}$ at the sample.

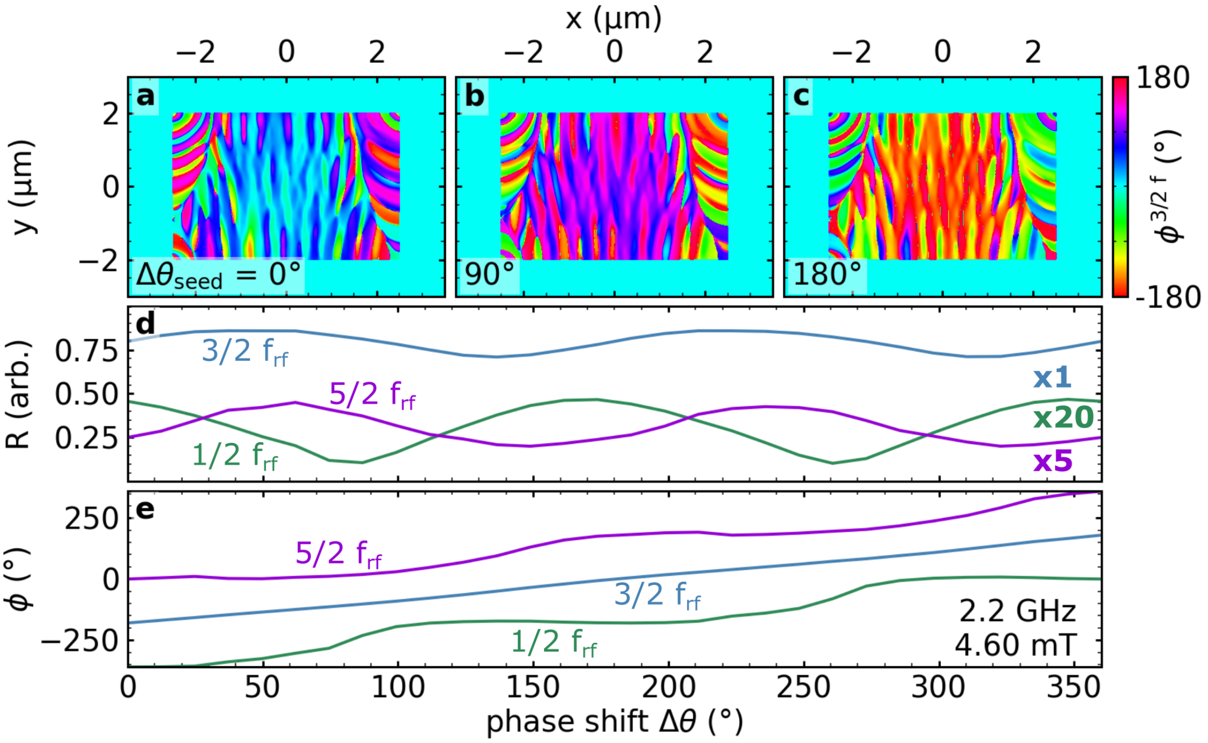

Fig. S11 | Micromagnetic simulation of the phase-locking of NLSWs to an external seed frequency. In the presented micromagnetic simulations we investigate the influence of an additional seed frequency at $3/2$ $\mathrm{f_{rf}}$ with an amplitude of $0\,\mathrm{dBm}$. We performed the simulations for a $5\,\mu\mathrm{m} \times 4\,\mu\mathrm{m}$ rectangular shaped element with magnetic properties of $Ni_{80}Fe_{20}$ and analyzed the non-linear response of the system as a function of the phase shift $\Delta\theta$. Panel **a-c** show spatially-resolved images of the NLSW signal's phase obtained at $3/2$ $\mathrm{f_{rf}}$ for three different phase shifts $\Delta\theta$. One clearly observes a reversal of the signal's sign from **a** to **c**. In analogy to our experiments, we analyzed the phase and the magnitude as a function of the phase shift $\Delta\theta$ in **e** and **f**. Here, the responses at $1/2$ $\mathrm{f_{rf}}$ (green), $3/2$ $\mathrm{f_{rf}}$ (blue) and $5/2$ $\mathrm{f_{rf}}$ (violet) are shown, respectively. In panel **d**, the normalized magnitude shows the expected $180°$ periodicity for all analyzed channels (scaling factors on the right). While the phase for the $3/2$ $\mathrm{f_{rf}}$ signal in **e** appears mostly linear, the phase values for both other NLSW channels indicate the presence of two distinct phase states. The mostly linear behavior of the phase at $3/2$ $\mathrm{f_{rf}}$ is due to a superposition of the NLSW signal at this frequency and the direct excitation at the seed frequency with similar wavev ector.
